# Advances in Ommaya reservoir–based intrathecal therapy for leptomeningeal metastasis from non–small cell lung cancer: a systematic review

**DOI:** 10.3389/fonc.2026.1746953

**Published:** 2026-05-19

**Authors:** Cai Jiang, Yongzhi Zhao, Daohui Chen, Xiaofeng Yuan

**Affiliations:** 1 Department of Neurosurgery, Quzhou Kecheng People’s Hospital, Quzhou, China; 2 Department of Neurosurgery, Haining Central Hospital, Hangzhou, China; 3 Quanzhou Mingzhou Rehabilitation Hospital, Quanzhou, China; 4 Ningbo Beilun Orthopedic Hospital, Ningbo, China

**Keywords:** complications, intrathecal chemotherapy, leptomeningeal metastasis, non–small cell lung cancer, Ommaya reservoir, targeted therapy

## Abstract

**Background:**

Leptomeningeal metastasis (LM) from non–small cell lung cancer (NSCLC) is a devastating complication associated with a poor prognosis, with historical median overall survival (OS) of approximately 3–6 months. Limited penetration of systemic therapies across the blood–brain and blood–cerebrospinal fluid (CSF) barriers has prompted increasing interest in intrathecal (IT) drug delivery. The Ommaya reservoir, an implantable ventricular access device, provides a practical route for achieving therapeutic drug concentrations within the CSF and has been increasingly applied in NSCLC-related LM.

**Objective:**

To systematically review contemporary evidence regarding the efficacy, safety, and delivery-route considerations of intrathecal (IT) therapy in NSCLC-related LM, with particular emphasis on the clinical role of Ommaya reservoir–based delivery.

**Methods:**

A comprehensive literature search of PubMed, MEDLINE, Embase, Scopus, Web of Science, the Cochrane Library, and CNKI was conducted from database inception to September 30, 2025. Original studies reporting clinical outcomes of intrathecal chemotherapy and/or targeted therapy in adult patients with NSCLC-related LM were reviewed in accordance with PRISMA 2020 guidelines. Delivery route (Ommaya reservoir vs lumbar puncture) was extracted when reported. Studies without extractable NSCLC-LM–specific clinical outcome data and narrative reviews/guidelines were excluded from the primary clinical efficacy synthesis and used for contextual background only.

**Results:**

Ten original studies met predefined criteria for NSCLC-specific clinical efficacy synthesis. Across these studies, intrathecal therapy was associated with neurological improvement and CSF cytology clearance in a substantial proportion of patients. Traditional IT agents such as methotrexate or cytarabine were generally associated with modest survival outcomes, whereas more recent studies evaluating IT pemetrexed and molecularly guided regimens reported longer survival in selected cohorts, particularly in EGFR-mutant NSCLC-LM. Studies using Ommaya reservoir–based delivery highlighted practical advantages in repeated CSF access and treatment continuity, while device-related complications (e.g., infection and catheter malfunction) were generally manageable. However, most available evidence derives from retrospective, single-center cohorts with heterogeneous treatment protocols, and randomized comparative evidence remains limited.

**Conclusion:**

Intrathecal therapy represents an important component of multimodal management for selected patients with NSCLC-related LM. Ommaya reservoir–based delivery may offer practical advantages for repeated treatment and CSF monitoring in appropriately selected patients, with acceptable toxicity and manageable device-related risks. Emerging data on pemetrexed-based intrathecal regimens and other molecularly informed approaches suggest potential benefit in selected subgroups, but prospective, multicenter, mutation-stratified studies are needed to refine patient selection, optimize dosing strategies, and define the comparative role of different intrathecal delivery routes.

## Introduction

Non–small cell lung cancer (NSCLC) accounts for approximately 85% of all lung cancer cases and remains a leading cause of cancer-related mortality worldwide (1). Leptomeningeal metastasis (LM), defined as the dissemination of malignant cells into the leptomeninges and cerebrospinal fluid (CSF), occurs in approximately 3-5% of patients with advanced NSCLC, with a higher reported incidence among patients harboring epidermal growth factor receptor (EGFR) mutations and, less commonly, anaplastic lymphoma kinase (ALK) rearrangements (2–4). Clinically, LM is characterized by debilitating symptoms such as headache, nausea, cranial neuropathies, gait disturbance, and other focal or diffuse neurological deficits, and is associated with a dismal prognosis. In historical NSCLC-LM cohorts, median overall survival (OS) typically ranges from 1.5 to 6 months in the absence of effective intervention (2, 3, 5). The limited ability of systemic chemotherapy and many targeted agents to traverse the blood–brain barrier and blood–CSF barrier represents a major obstacle to durable disease control in this setting, motivating increasing interest in intrathecal (IT) drug delivery (2, 6, 7). The Ommaya reservoir, introduced by Ayub Ommaya in the 1960s, is an implantable ventricular access device that permits repeated administration of therapeutic agents directly into the CSF, thereby bypassing these barriers (8). The system consists of a subcutaneous dome connected to a ventricular catheter, enabling reliable access for IT chemotherapy, targeted agents, and CSF sampling/monitoring (8, 9). In NSCLC-related LM, intrathecal treatment has historically included methotrexate (MTX) and cytarabine-based regimens, while more recent studies have increasingly evaluated pemetrexed-based intrathecal strategies in molecularly selected populations (3, 10–18). Importantly, current evidence does not demonstrate clear superiority of Ommaya-based administration over serial lumbar puncture in terms of treatment efficacy, and direct comparative evidence remains limited (13, 14). Rather than representing an independent therapeutic breakthrough, the Ommaya reservoir primarily facilitates consistent CSF drug exposure, repeated administration, and improved procedural tolerability, particularly in the context of modern intrathecal regimens (8, 9, 11, 12). In molecularly defined subgroups—particularly EGFR-mutant NSCLC—combining intrathecal therapy with systemic tyrosine kinase inhibitors (TKIs) has emerged as a promising strategy to enhance intracranial disease control (10–18). Evidence regarding LM management in ALK-rearranged NSCLC remains more limited and is often derived from small or heterogeneous cohorts, with many reports emphasizing systemic targeted approaches rather than standardized intrathecal protocols (4, 19, 20). Despite these advances, intrathecal treatment in NSCLC-LM remains accompanied by important challenges. Device-related complications associated with Ommaya reservoir use, including infection, catheter obstruction, CSF leakage, and procedure-related morbidity, may offset clinical benefits if not carefully prevented and managed (21–24). In parallel, the absence of standardized IT dosing schedules, heterogeneity in concomitant systemic treatments, and the scarcity of large prospective comparative studies hinder the development of evidence-based treatment algorithms (2, 7, 13–18). Technical refinements such as neuronavigation- and endoscopy-assisted Ommaya placement may improve procedural accuracy and safety in selected settings (25–28). Against this background, an updated synthesis of the literature is needed to clarify clinical outcomes of intrathecal therapy in NSCLC-LM while defining the practical role of Ommaya reservoir–based delivery within multimodal management. The present systematic review aims to summarize current evidence regarding efficacy, safety profiles, delivery-route considerations, and technical innovations related to intrathecal therapy for NSCLC-LM, with particular emphasis on Ommaya reservoir use, and to highlight key knowledge gaps and priorities for future research. The review was conducted in accordance with the Preferred Reporting Items for Systematic Reviews and Meta-Analyses (PRISMA 2020) guidelines to ensure methodological rigor and transparency (29).

## Methods

### Study design and protocol

This study was conducted as a systematic review following the Preferred Reporting Items for Systematic Reviews and Meta-Analyses (PRISMA 2020) statement (29). Although the protocol was not prospectively registered in PROSPERO, the methodology was predefined and adhered closely to PRISMA 2020 recommendations to ensure transparency and reproducibility. The primary objective was to evaluate the clinical application, efficacy, safety, and technical advances of intrathecal (IT) therapy in the management of leptomeningeal metastasis (LM) from non–small cell lung cancer (NSCLC), with particular emphasis on the role of Ommaya reservoir–based delivery and delivery-route considerations.

### Research question and PICO framework

The review question was defined using the PICO framework:

- Population (P): Adults (≥18 years) diagnosed with NSCLC-related LM confirmed by CSF cytology and/or neuroimaging.
- Intervention (I): IT administration of antitumor agents (chemotherapy or targeted drugs) for NSCLC-LM, delivered via an Ommaya reservoir and/or lumbar puncture.
- Comparison (C): Different IT delivery routes (Ommaya reservoir vs lumbar puncture, when available), systemic therapy, or best supportive care.
- Outcomes (O): Overall survival (OS), progression-free survival (PFS), neurological symptom improvement, CSF cytology clearance, complication rates, and pharmacokinetic profiles.

### Search strategy

A comprehensive literature search was performed in PubMed, MEDLINE, Embase, Scopus, Web of Science, the Cochrane Library, and CNKI from database inception to September 30, 2025. Search terms combined controlled vocabulary (MeSH/Emtree) and free-text keywords, for example: (“Ommaya reservoir” OR “intraventricular access device” OR “intrathecal catheter” OR “lumbar puncture”) AND (“leptomeningeal metastasis” OR “meningeal carcinomatosis” OR “CSF metastasis”) AND (“non-small cell lung cancer” OR “NSCLC”). Boolean operators (“AND”, “OR”) and filters (human subjects, English language) were applied. Studies published in English or Chinese were considered. Reference lists of included articles and relevant reviews were also manually screened to identify additional eligible studies.

### Inclusion and exclusion criteria

Inclusion criteria:

1. Adult patients (≥18 years) with cytologically and/or radiologically confirmed NSCLC-LM.
2. Intervention involving IT drug delivery (chemotherapy and/or targeted therapy) for NSCLC-LM, with the delivery route specified or inferable (Ommaya reservoir, lumbar puncture, or mixed approaches).
3. Reporting at least one clinical outcome (OS, PFS, neurological improvement, CSF clearance, pharmacokinetic data, or safety data).
4. Original articles in English or Chinese, including randomized controlled trials (RCTs), prospective or retrospective cohort studies, and case series with ≥3 patients.

Exclusion criteria:

1. Non-human or pediatric studies.
2. LM of non-NSCLC origin.
3. Studies of systemic therapy alone without an intrathecal treatment component.
4. Reviews, editorials, guidelines, and conference abstracts without extractable outcome data for NSCLC-LM patients receiving IT therapy.
5. Duplicated or overlapping cohorts (in such cases, the most informative or most recent dataset was retained).

### Study selection

**Figure 1:**
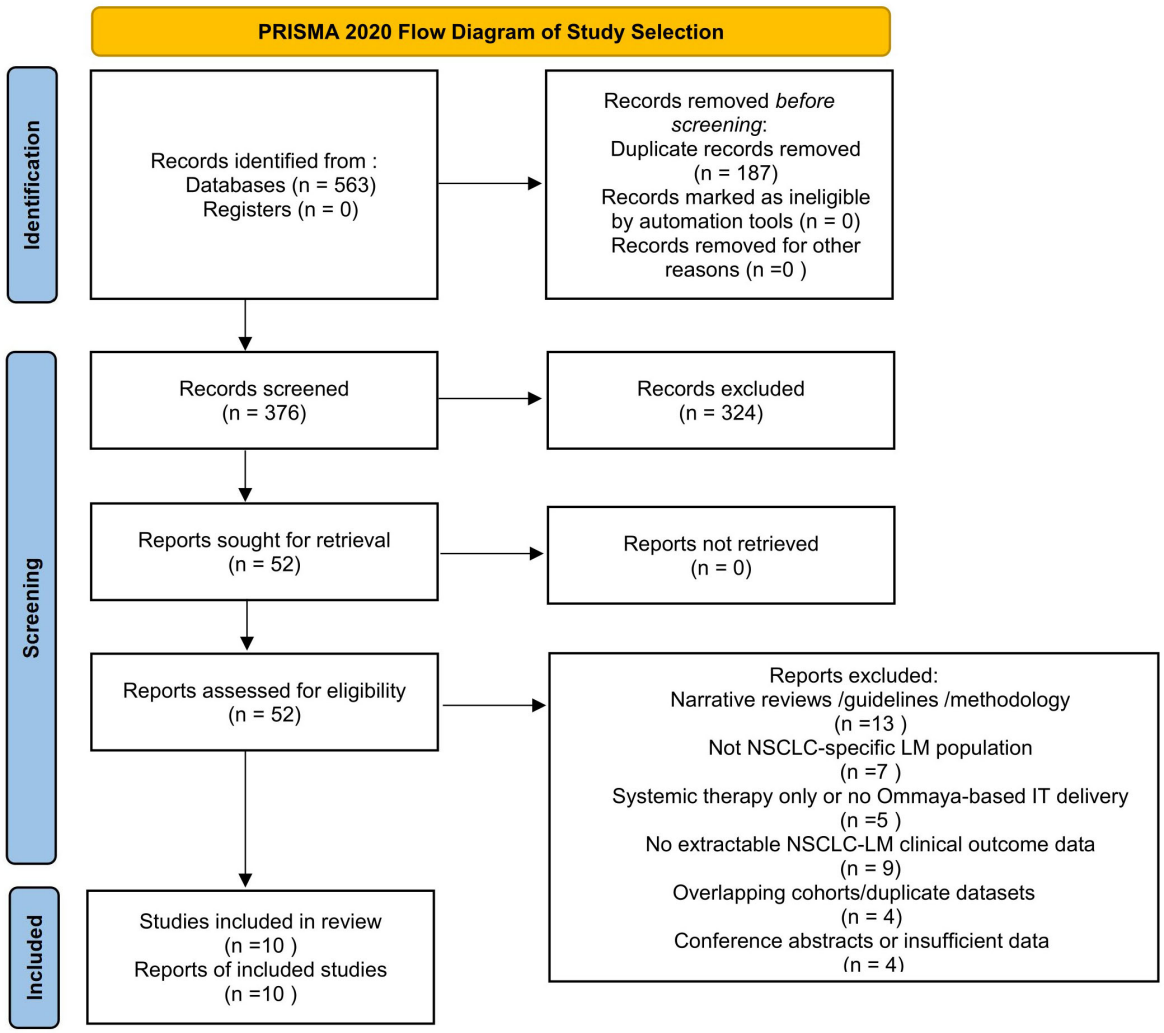
PRISMA 2020 flow diagram.

All retrieved records were imported into EndNote X9 for duplicate removal. Two reviewers independently screened titles and abstracts, followed by full-text assessment based on predefined inclusion and exclusion criteria. Disagreements were resolved through discussion with a third reviewer. The study selection process is summarized in a PRISMA 2020–compliant flow diagram (**Figure 1**). During revision, all included studies and citation metadata (PMID/DOI) were re-verified to ensure accuracy and reproducibility.

### Data extraction

Two reviewers independently extracted data using a standardized form, including author, publication year, country, study design, sample size, patient characteristics, median age, mutation status (EGFR/ALK/wild type, when available), IT delivery route (Ommaya reservoir, lumbar puncture, or mixed), intrathecal agent, IT regimen and dosing frequency, combination therapy, clinical outcomes (OS, PFS, neurological response/improvement, CSF cytology clearance, complications), pharmacokinetic findings, and follow-up duration. Studies providing extractable NSCLC-specific clinical data on IT therapy were included in the primary qualitative synthesis and summarized in **Table 1**, with delivery route explicitly recorded. Additional original studies, narrative reviews, and guidelines that did not meet criteria for the primary synthesis were used for contextual background and discussion only.

**Table 1:** Clinical studies of intrathecal chemotherapy in NSCLC-related leptomeningeal metastasis, with delivery route specified (Ommaya reservoir vs lumbar puncture).

Ref	Study (author,year)	Country	Study design	NSCLC-LM patients (n)	Median age (yrs)	Mutation (EGFR/ALK/WT)	Delivery route	Intrathecal agent	IT regimen	Combination therapy	Median OS (mo)	Main findings	Reference
3	Gwak HS, 2013	Korea	Retrospective cohort	105	56	NR	Ommaya reservoir	MTX/Ara-C based	Median 5 rounds (range 1–49)	Concurrent systemic chemotherapy (improved OS); aggressive ICP control	3	Symptom response modest (headache response 42%); CSF cytology negative conversion 7.6%; OS 3.0 mo.	(3)
8	Li M, 2026	China	Reprospective real-world cohort study	200	56	EGFR-mutant	Ommaya reservoir	Pemetrexed	10-20mgweekly*4,then q2w*2months,then monthly maintenance	Whole-brain radiotherapy (WBRT)	Overall cohort: 12.3 months IT + WBRT group: 18.5 months No local therapy: 8.8 months	Combined IT pemetrexed via Ommaya + WBRT significantly improved OS ECOG score and third-generation TKI were independent prognostic factors Acceptable safety profile	(10)
9	Li H, 2023	China	Prospective single-arm phase I trial	23	55	EGFR-mutant and ALK	Ommaya reservoir	Pemetrexed	30–50 mg on Days 1 & 8 every 3 weeks; RD = 30 mg	Prior ≥2 treatments; systemic therapy NR in abstract	9.5	ORR 43.5%; DCR 82.6%; median PFS 6.3 mo; manageable toxicity; PK favors Ommaya vs LP.	(11)
10	Wang Z, 2025	China	Retrospective cohort + propensity score matching	157 (54 IP; 103 non-IP); matched 41:41	≤60 years 73.2%, >60 years 26.8%.	EGFR-mutant predominant (Exon 19 47.6%, Exon 20 39.0%, Exon 21 4.9%); EGFR-negative 8.5%.	Ommaya reservoir	Pemetrexed	20–50 mg; initially 1–2×/week, then maintenance q3 weeks	Systemic TKIs/chemo/radiotherapy as clinically indicated	11.2	IP improved OS vs non-IP (11.2 vs 5.1 mo after PSM); benefit notable in ECOG 3–4; exploratory CSF/plasma biomarkers.	(12)
17	Fan C, 2024	China	Prospective phase II	132	52	EGFR-mutant	Lumbar puncture/Ommaya (9 cases)	Pemetrexed	50 mg d1,d5 then q3w ×4cycles then monthly maintenance	Systemic EGFR-TKI	12.0	RANO response 80.3%; no OS difference by route; manageable toxicity	(13)
19	Zhang TL, 2025	China	Retrospective cohort	104	56	EGFR-mutant	Lumbar puncture (72.1%); Ommaya (27.9%)	Pemetrexed	20-50 mgDay 1 & Day 8, q4 weeks	Double-dose 3rd-gen EGFR-TKI; Bevacizumab (42.3%); some chemo/RT	13.0	ORR 77.9%; myelosuppression 58.7%; Bevacizumab improved OS; no OS difference between Ommaya vs LP	(14)
20	Geng, 2022	China	Retrospective study	34	54	EGFR-mutant 79.4%; ALK 11.8%; WT 5.9%; Unknown 2.9%	Lumbar puncture	Pemetrexed	15-40 mg; twice weekly (3-4 days interval); median 3 administrations (range 1-12)	Systemic therapy (EGFR-TKI/chemotherapy/ALK inhibitor individualized)	20	Neurological improvement 76.5%; manageable toxicity; no grade 3-4 adverse events; median OS 20 months	(15)
21	Noronha V, 2024	India	Retrospective cohort	16	NR	EGFR-mutant 13/16 (81.3%	Lumbar puncture	Pemetrexed	50 mg per administration (schedule NR)	Concurrent systemic therapy in 15/16 (TKI and/or chemotherapy); prior RT 75%	7.5	Median OS from LM diagnosis 7.5 mo (95% CI 1.2-13.8); OS from start of IT 2.7 mo; manageable toxicity; delays due to cytopenia.	(16)
22	Miao Q, 2020	China	Retrospective cohort	23	53	EGFR 69.6%; ALK 8.7%; ROS1 4.3%; ERBB2 4.3%; WT 13.0%	Lumbar puncture ± Ommaya reservoir	Pemetrexed + dexamethasone	10 mg weekly (until CSF negative ×2/progression/intolerance)	Systemic TKI/chemotherapy/anti-angiogenesis ± WBRT	Not mature	Median PFS 9.6 mo; ORR 34.8%; manageable toxicity; IP-based multimodal therapy effective in refractory LM.	(17)
23	Pan Z, 2019	China	Prospective phase I trial	13	55	EGFR-mutant predominant; 1 ALK-positive	Lumbar puncture	Pemetrexed	10-15 mg; twice weekly ×2 weeks (induction), then weekly ×4 weeks (consolidation); MTD 10 mg	Prior radiotherapy and systemic therapy allowed; some continued EGFR-TKI	3.5	MTD 10 mg; ORR 31%; DCR 54%; manageable toxicity with vitamin B12/folate supplementation; hematologic toxicity dose-limiting	(18)

### Definitions of outcomes

Given the heterogeneity in outcome reporting across included studies, predefined operational definitions were applied where possible. Neurological improvement was defined as partial or complete improvement of leptomeningeal metastasis–related neurological symptoms, as reported by the original study authors. CSF clearance was defined as conversion from positive to negative cerebrospinal fluid cytology following intrathecal therapy, when reported in the primary study. When studies used alternative terminology or did not provide explicit definitions, outcomes were interpreted according to the authors’ original descriptions.

### Quality assessment

The methodological quality of the included original clinical studies was assessed descriptively. Observational studies were evaluated using the Newcastle–Ottawa Scale (NOS) (30), and prospective or interventional studies were assessed using the Cochrane Risk of Bias 2.0 (RoB2) tool (31). Given the heterogeneity of study designs and outcomes, results of the quality assessment are summarized narratively in the text rather than presented in a separate table.

### Data synthesis and analysis

Given the substantial heterogeneity in study designs, patient populations, IT agents/regimens, delivery routes, and outcome definitions, a qualitative synthesis rather than a formal meta-analysis was performed. Findings were summarized narratively, focusing on treatment efficacy, safety/complication profiles, delivery-route considerations (Ommaya reservoir vs lumbar puncture vs mixed approaches), mutation-specific responses (particularly EGFR-mutant disease), and technical advances such as neuronavigation- or endoscopy-assisted Ommaya implantation. Where available, ranges and confidence intervals reported in primary studies were incorporated to provide quantitative context. Because most included studies were non-randomized and route selection was influenced by clinical factors, no causal inference regarding superiority of one delivery route over another was made.

## Results

### Study selection

A total of 563 records were retrieved from seven electronic databases. After removal of 187 duplicates, 376 unique records were screened by title and abstract, and 52 full-text articles were assessed for eligibility. Following full-text review, 25 articles were excluded, including narrative reviews/guidelines/methodological papers (n = 13), studies without NSCLC-specific LM populations (n = 7), and studies of systemic therapy alone or without extractable intrathecal treatment data (n = 5). Among the remaining original studies, 10 studies met the predefined criteria for the primary qualitative synthesis, defined as reporting patients with leptomeningeal metastasis from non–small cell lung cancer (NSCLC-LM) treated with intrathecal (IT) therapy and providing extractable clinical outcomes, with delivery route specified (Ommaya reservoir, lumbar puncture, or mixed approaches). These 10 studies were included in the final clinical synthesis and are summarized in **Table 1**. Additional original studies were reviewed to provide contextual information on device-related safety, technical considerations, and procedural advances, but were not used to infer NSCLC-LM–specific efficacy outcomes. The study selection process is illustrated in **Figure 1** (PRISMA 2020 flow diagram).

### Study characteristics

The 10 included studies, published between 2013 and 2025, provided original clinical evidence on intrathecal therapy for NSCLC-related leptomeningeal metastasis, with delivery route explicitly specified in **Table 1** (Ommaya reservoir, lumbar puncture, or mixed routes). Study designs included retrospective observational cohorts, prospective early-phase clinical studies, and matched survival analyses. Intrathecal agents evaluated across studies included methotrexate, cytarabine, pemetrexed, and osimertinib, often administered in combination with contemporary systemic therapies and, in selected cohorts, radiotherapy. Baseline demographic and molecular characteristics were extracted when explicitly reported; variables not provided in the original publications were recorded as not reported. A detailed summary of study characteristics and design features is presented in **Table 1**.

### Survival outcomes

**Figure 2:**
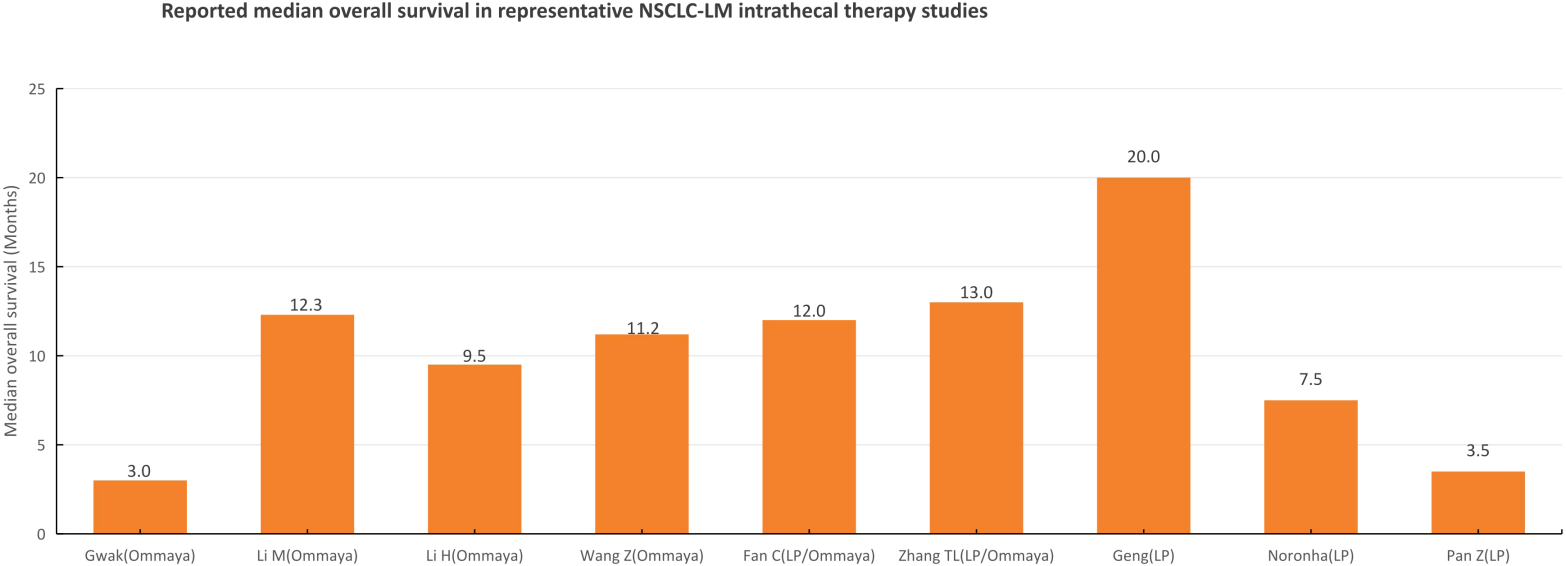
Illustrative summary of reported overall survival outcomes in representative studies of intrathecal therapy for NSCLC-related leptomeningeal metastasis, with delivery route specified (Ommaya reservoir, lumbar puncture, or mixed approaches). The bar chart displays reported median overall survival values from selected retrospective cohorts and clinical studies. Data are presented for descriptive purposes only and do not represent a pooled or comparative analysis. Survival outcomes vary substantially according to molecular subtype, performance status, intrathecal regimen, delivery route, and concomitant systemic/radiation therapy (3, 10–18).

Reported overall survival (OS) outcomes varied substantially across the included studies, reflecting heterogeneity in patient selection, performance status, molecular subtype, intrathecal agents, delivery route, and multimodal treatment strategies. In a large retrospective cohort of 105 NSCLC-LM patients receiving intraventricular chemotherapy, Gwak et al. reported a median OS of 3.0 months (3). More recent studies evaluating intrathecal pemetrexed (via Ommaya reservoir, lumbar puncture, or mixed delivery strategies depending on study design) reported improved outcomes in selected patient populations (10–18). In prospective and retrospective cohorts enriched for EGFR-mutant disease and treated with contemporary systemic TKIs, median survival was generally longer than historical controls, although cross-study comparisons should be interpreted cautiously because of differences in eligibility criteria, route selection, concomitant therapies, and outcome definitions (10, 13, 14, 16–18). In the propensity score–matched analysis by Wang et al., intrathecal pemetrexed was associated with improved survival compared with non-intrathecal treatment in selected patients, including a notable benefit in patients with poor performance status (12). Overall, representative NSCLC-LM studies suggest that IT therapy may provide clinical benefit in carefully selected patients, while the specific contribution of delivery route (Ommaya reservoir vs lumbar puncture) remains difficult to isolate from regimen effects and patient selection. Because of substantial clinical and methodological heterogeneity, a formal meta-analysis was not performed. Survival outcomes from representative studies are illustrated in **Figure 2** and described qualitatively in the text for descriptive comparison only, given the substantial heterogeneity in patient populations, delivery routes, and concomitant treatments.

### Safety and complications

**Figure 3:**
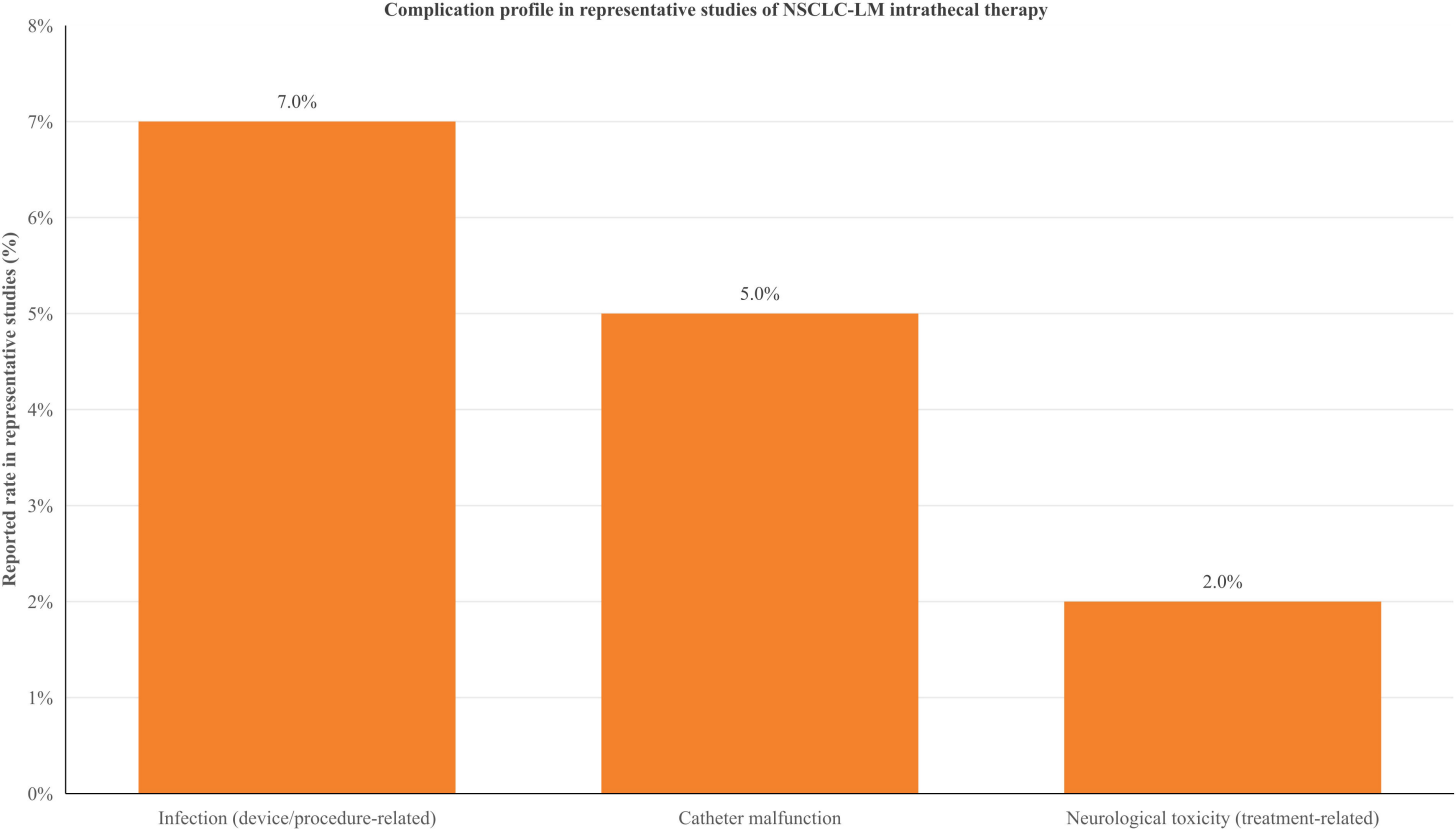
Illustrative summary of reported complication categories in representative studies of intrathecal therapy and Ommaya-related procedures for NSCLC-related leptomeningeal metastasis. The bar chart presents representative reported rates of selected adverse events (infection, catheter malfunction/obstruction, and neurologic toxicity) extracted from studies with heterogeneous populations and treatment protocols. These values are shown for descriptive visualization only and do not represent pooled estimates or a formal comparative analysis (15, 16, 21–24, 32, 33).

Device-related and treatment-related adverse events were evaluated across original studies reporting safety outcomes. Reported complications included infectious events, catheter-related malfunction/obstruction (in studies involving Ommaya reservoir placement), and treatment-related toxicities such as myelosuppression or neurotoxicity, depending on the intrathecal agent and dosing schedule (15, 16, 18, 21–23, 32, 33). Although device-related complications were clinically relevant and required prompt recognition and management, most reports described manageable toxicity profiles when appropriate monitoring and supportive care were used. Because a substantial proportion of Ommaya-specific safety data were derived from mixed-tumor cohorts and technical/device-focused series rather than NSCLC-specific efficacy studies, these findings were interpreted as contextual device-related evidence rather than NSCLC-specific treatment efficacy outcomes. Selected complication categories reported in representative studies are illustrated in **Figure 3** and summarized descriptively in the text for contextual interpretation only, as safety data were derived from heterogeneous cohorts and were not synthesized quantitatively.

### Technical success and procedural advances

**Figure 4:**
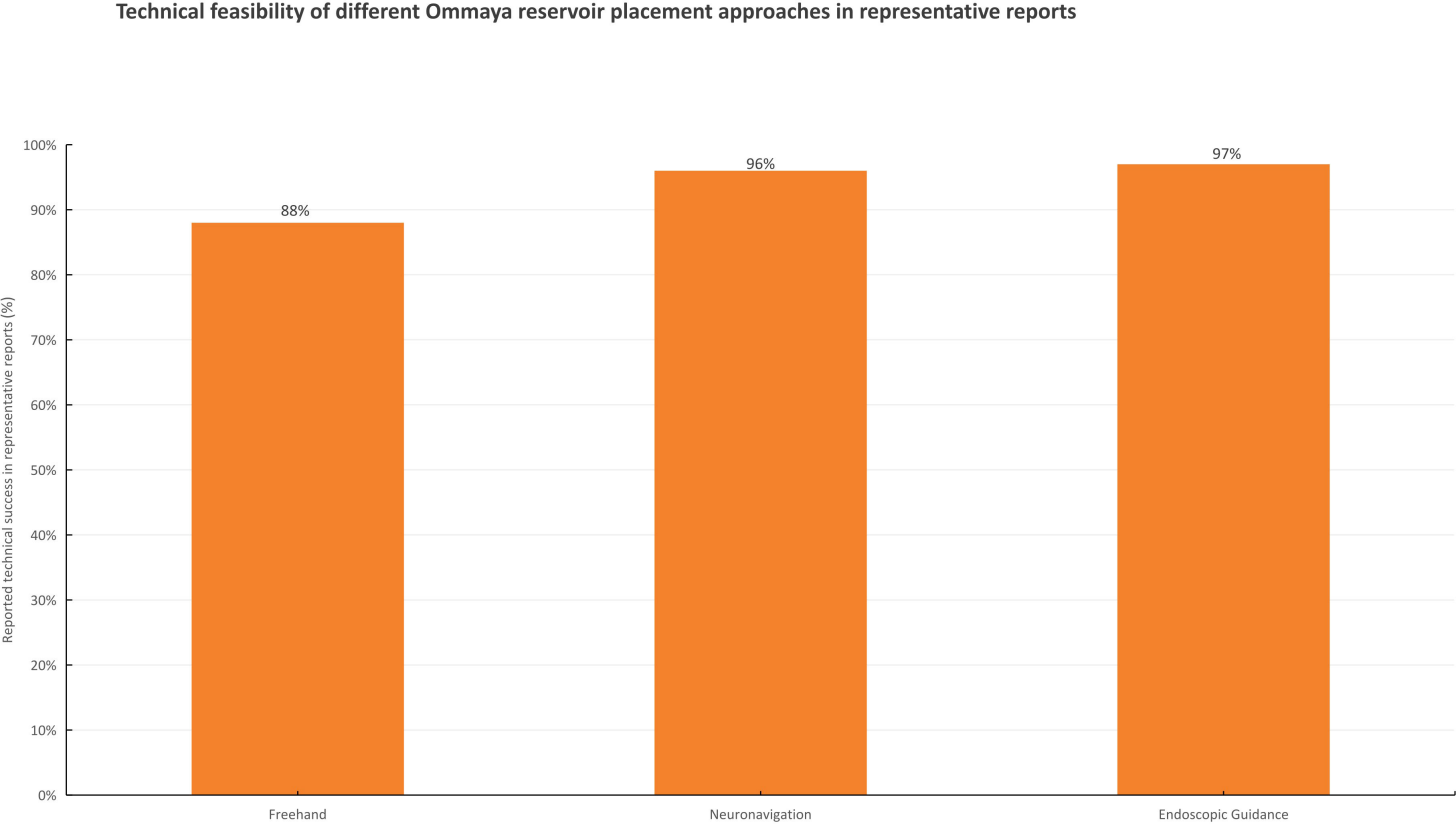
Illustrative summary of reported technical success associated with different approaches to Ommaya reservoir placement. The chart presents representative reported success rates for freehand, neuronavigation-assisted, and endoscopic-guided placement from heterogeneous technical studies. Values are shown for descriptive purposes only and do not represent pooled estimates or formal comparative analysis. Technical success was defined according to the original reports (e.g., accurate catheter placement and/or absence of immediate procedural complications) (25–28).

Several studies have described technical refinements in Ommaya reservoir placement, including neuronavigation-assisted and endoscopic-guided approaches (25–28). These reports generally support the feasibility and procedural applicability of image-guided techniques in representative cohorts. However, most available data are retrospective, and direct head-to-head comparisons with conventional freehand placement are limited. Therefore, the reported technical feasibility and placement success metrics are presented descriptively in **Figure 4** rather than as definitive evidence of superiority.

### Trends in research output

**Figure 5:**
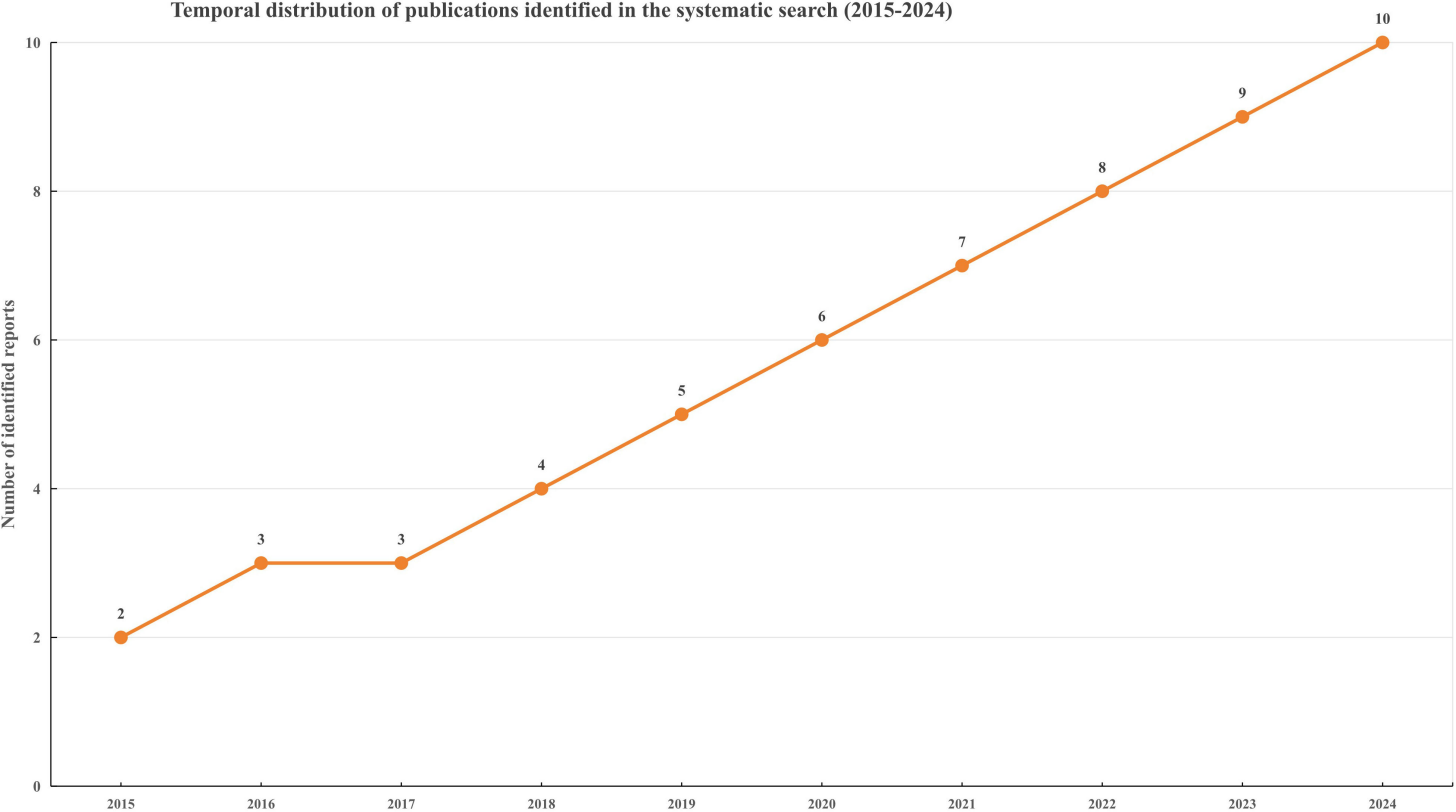
Temporal distribution of publications identified in the systematic literature search (2015-2024). The line chart shows the annual number of reports identified by the predefined search strategy and included for screening. The temporal pattern reflects research activity and publication capture within the search framework, and should not be interpreted as a formal bibliometric analysis (29).

The number of reports identified in the predefined systematic search showed an overall increasing trend across the study period. This pattern suggests growing research attention to intrathecal treatment strategies and Ommaya reservoir–related procedural issues in leptomeningeal metastasis. Because these counts are derived from the review search process rather than a formal bibliometric analysis, they are presented for descriptive context only. The temporal distribution of identified reports is illustrated in **Figure 5**.

### Quality assessment

Quality assessment using the Newcastle–Ottawa Scale (NOS) and the Cochrane Risk of Bias 2 (RoB 2) tool indicated that most observational studies were of moderate methodological quality, primarily due to retrospective design, single-center settings, and limited sample sizes (30, 31). Prospective and multicenter investigations provided relatively more robust estimates of safety and efficacy, although heterogeneity in outcome definitions and treatment protocols remained substantial. Given the diversity of study designs and reporting standards, quality assessment findings were summarized narratively rather than presented in a separate table.

## Discussion

This systematic review provides an updated synthesis of the literature on intrathecal (IT) chemotherapy for leptomeningeal metastasis from non-small cell lung cancer (NSCLC-LM), with particular attention to the role of Ommaya reservoir–based delivery. The efficacy-focused synthesis was based on ten original studies with extractable NSCLC-LM–specific clinical outcomes, while additional original studies were reviewed to contextualize device-related safety, complications, and technical evolution. Together, these data provide a pragmatic overview of treatment outcomes, route-specific procedural considerations, and emerging multimodal strategies relevant to IT therapy in NSCLC-LM.

### Comparison with previous evidence

Earlier reviews of leptomeningeal metastasis largely emphasized systemic therapies, general LM management principles, or pharmacologic strategies, with relatively limited focus on intrathecal access routes and delivery platforms (2, 6, 7, 34). In contrast, the present review specifically examined NSCLC-LM studies of IT therapy and incorporated route information (Ommaya reservoir vs lumbar puncture) when reported. Across the included literature, Ommaya-based delivery appears to facilitate repeated administration and consistent CSF access, while lumbar puncture remains widely used and clinically practical in many settings. Current evidence does not establish clear superiority of one route over another in terms of survival outcomes across all patient populations, but route selection may influence treatment feasibility, tolerability, and continuity in real-world practice (13, 14). Historically, outcomes with traditional IT agents such as methotrexate or cytarabine were modest in NSCLC-LM cohorts (3). More recent studies of intrathecal pemetrexed-based regimens, particularly in EGFR-mutant populations and in combination with contemporary systemic therapy, have reported improved outcomes in representative cohorts (10–18). However, cross-study comparisons should be interpreted cautiously because of substantial heterogeneity in patient selection, molecular subtype, performance status, concomitant therapies, and outcome definitions.

### Comparative effectiveness and safety

The present review supports the clinical feasibility of IT therapy in selected NSCLC-LM patients and highlights the practical role of Ommaya reservoir–mediated delivery in patients requiring repeated administrations. Reported infection rates and catheter malfunction rates in Ommaya-related series were generally within the range reported for implantable CSF access devices in neuro-oncology practice (21–24, 32). At the same time, a portion of the safety literature is derived from mixed-tumor cohorts rather than NSCLC-only populations; these data should therefore be interpreted as contextual device-related evidence rather than disease-specific efficacy evidence. Drug-related adverse events remain an important consideration. Methotrexate has been associated with neurotoxicity in some settings, whereas pemetrexed-based regimens are more commonly associated with hematologic toxicity and transient meningeal irritation/chemical meningitis, depending on dose, schedule, and supportive measures (13–18, 33). In modern series, most adverse events were manageable with dose adjustment, vitamin supplementation, corticosteroid co-medication, and supportive care (13, 15, 17, 18). Preventive strategies—including meticulous aseptic technique, standardized catheter care, and careful monitoring during repeated IT administration—remain essential to minimize complications and maintain treatment continuity.

### Hydrocephalus and CSF dynamics

Hydrocephalus is a common and clinically relevant complication in leptomeningeal metastasis, regardless of primary tumor histology. Altered CSF flow dynamics may impair intrathecal drug distribution and reduce therapeutic effectiveness. Therefore, assessment of CSF circulation, intracranial pressure, and disease distribution should be incorporated into treatment planning when considering IT therapy, particularly for patients being evaluated for Ommaya reservoir placement or continued ventricular administration.

### Evidence level and research gaps

Most available evidence remains retrospective, single-center, and heterogeneous in methodology, with only a limited number of prospective or matched analyses (10–18). This limits causal inference and precludes robust quantitative comparison across IT regimens and delivery routes. In addition, outcome definitions (e.g., neurological response, CSF cytology clearance), timing of assessment, and concomitant systemic treatment strategies vary substantially across studies, complicating pooled interpretation. Clinical outcomes appear to differ by molecular subtype, systemic treatment context, and performance status. In particular, EGFR-mutant NSCLC-LM populations have shown encouraging outcomes in several pemetrexed-based IT studies conducted in the era of EGFR-TKIs (10–18). Future studies should prospectively stratify patients by molecular profile, prior systemic therapy exposure, CSF flow status, and delivery route (Ommaya reservoir vs lumbar puncture), and should incorporate standardized endpoints to better identify subgroups most likely to benefit from IT therapy.

### Mechanistic and translational considerations

From a mechanistic perspective, the efficacy and toxicity of IT therapy are influenced by CSF flow dynamics, neuraxial drug distribution, local tumor burden, and pharmacokinetic/pharmacodynamic (PK/PD) relationships. These considerations are particularly relevant when comparing ventricular delivery through an Ommaya reservoir with lumbar administration, as route-dependent differences in CSF distribution and exposure may affect both therapeutic activity and tolerability. Integration of PK/PD-informed dosing strategies into prospective protocols may help optimize schedules and improve therapeutic indices in future studies (11–13, 18, 35).

### Technical and procedural evolution

Advances in Ommaya reservoir implantation techniques have paralleled broader developments in neurosurgical precision. Neuronavigation-assisted and endoscopic-guided approaches have been increasingly adopted to improve catheter placement accuracy and procedural reliability in representative cohorts (25–28). However, because available studies are mostly retrospective and differ in design, operator experience, and outcome definitions, these data should be interpreted as descriptive technical evidence rather than definitive comparative-effectiveness evidence. Future studies using standardized procedural endpoints and complication reporting may clarify the incremental value of image-guided implantation approaches.

### Clinical implications and multimodal management

Intrathecal therapy has become an important component of multimodal management for selected patients with NSCLC-LM, particularly when integrated with contemporary systemic treatment and supportive neurosurgical care. Ommaya reservoir–based delivery is especially relevant in patients requiring repeated administration, prolonged treatment courses, or improved procedural tolerability, while lumbar puncture remains a practical option in many clinical settings. Route selection should therefore be individualized according to patient condition, treatment goals, CSF dynamics, and institutional expertise. In parallel, stereotactic radiotherapy—particularly stereotactic radiosurgery (SRS) and hypofractionated SRS—remains an important option for carefully selected patients with focal or nodular CNS disease, including selected leptomeningeal presentations (36, 37). These approaches may complement IT and systemic therapies within multimodal treatment pathways. Multidisciplinary collaboration among neurosurgeons, medical oncologists, radiation oncologists, and neuroradiologists is essential to optimize sequencing and personalize treatment decisions.

### Limitations and future directions

This review has several limitations. First, most included studies were retrospective with relatively small sample sizes, increasing the risk of selection bias and confounding. Second, publication bias cannot be excluded. Third, substantial heterogeneity in IT agents, dosing schedules, delivery routes, concomitant systemic therapy, and outcome definitions limited direct comparability and precluded formal meta-analysis. Fourth, although PRISMA 2020 guidance was followed, the review protocol was not prospectively registered. In addition, future work should incorporate guideline-informed multimodal management and patient-centered outcomes (including quality of life) in NSCLC-LM care (5, 38–41). Future research should prioritize prospective, multicenter, mutation-stratified studies with standardized definitions of neurological response and CSF cytology clearance, route-stratified analyses (Ommaya reservoir vs lumbar puncture), PK/PD-guided dosing optimization, and systematic reporting of device-related and treatment-related adverse events. Such efforts are needed to define the precise role of Ommaya reservoir–based delivery within modern NSCLC-LM management.

## Conclusion

Intrathecal therapy is an important treatment component for selected patients with leptomeningeal metastasis from non-small cell lung cancer (NSCLC-LM), particularly when integrated into a multimodal treatment strategy. Ommaya reservoir–based delivery plays a practical role in facilitating repeated cerebrospinal fluid access and sustained intrathecal administration, while lumbar puncture remains a clinically relevant alternative in many settings. Current evidence suggests that intrathecal chemotherapy, especially pemetrexed-based regimens in contemporary treatment contexts, may provide meaningful clinical benefit in selected patient populations, with generally manageable toxicity. However, the available evidence remains heterogeneous and is derived predominantly from retrospective or early-phase studies, with limited direct comparisons across agents and delivery routes. Future research should prioritize prospective, multicenter, mutation-stratified studies with standardized outcome definitions, route-specific analyses (Ommaya reservoir vs lumbar puncture), and optimized dosing strategies to better define the role of intrathecal therapy—and the specific contribution of Ommaya reservoir–based delivery—in modern NSCLC-LM management.
